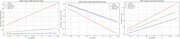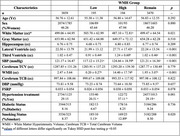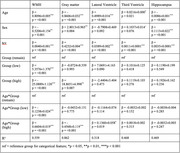# Evidence and Characteristics of Favorable Aging Group Within the Framingham Heart Study

**DOI:** 10.1002/alz70860_103888

**Published:** 2025-12-23

**Authors:** Addison Berg, Stephanie Sinclair, Cesar Moreno, Mae Yue So, Ana Nazmi Glosson, Ashley Acosta‐Parra, Charles S. DeCarli

**Affiliations:** ^1^ UC Davis, Davis, CA, USA; ^2^ IDeA Laboratory, Department of Neurology, UC Davis, Davis, CA, USA

## Abstract

**Background:**

White matter hyperintensities (WMH) on brain MRIs reflect tissue damage and are linked to cognitive decline and neurodegeneration. While studies have identified “normal” and “high” WMH accumulation in aging cohorts, literature on favorable aging subgroups and their health characteristics is limited. For this study, we hypothesize that individuals with below age, sex and head size‐expected WMH will have fewer vascular risk factors and less evidence of neurodegeneration suggesting better brain health.

**Methods:**

Data are from (*n* = 3859) Framingham Heart Study individuals with MRI, log normal transformed WMH volumes as dependent variables in a linear regression model of age, sex, and total cranial volume to create adjusted residuals. These residuals were then categorized as “low”, “high”, and “remaining” WMH groups based on the lowest 5% (*n* = 195), highest 5% (*n* = 194), and middle 90% (*n* = 3470) of volume, respectively. Separate regression models examined associations between age, sex, total cranial volume, group, and the interaction between age and group with MRI measures of brain atrophy. Additionally, between‐groups comparisons of vascular factors were performed.

**Results:**

As expected, the low group had smaller WMH volumes and limited age‐related increases, whereas the other groups had higher mean and significant age‐related increases in WMH volumes (Figure 1). The low group had lower mean ventricular volumes than the remaining and high groups. Separate regression modeling found significantly less age‐related gray matter volume loss compared to the high group (Table 2). The low group also had significantly lower systolic blood pressure, rates of hypertension, and Framingham Stroke Risk Profile Scores compared to the remaining and high groups (Table 1). These vascular factors were also associated with higher WMH and ventricle volumes, lower cerebral gray matter, and had significant interactions with subgroup designation.

**Conclusions:**

These findings indicate that a group with favorable brain outcomes may exist with low WMH, smaller average ventricle size, and less average neurodegeneration. One explanation for this group's favorable outcomes is their elevated vascular health, which has been closely tied to healthy brain aging.